# Trends in Incidence and Survival of Patients with Pancreatic Neuroendocrine Neoplasm, 1987–2016

**DOI:** 10.1155/2021/4302675

**Published:** 2021-12-22

**Authors:** Jingxuan Wang, Jianhua Liu, Chao He, Tiantian Sun, Yan Yan, Gang Che, Xuemin Li, Huanhuan Sun, Haiqing Ma

**Affiliations:** ^1^Department of Oncology, The Fifth Affiliated Hospital of Sun Yat-Sen University, Zhuhai, Guangdong, China; ^2^Department of Oncology, Guangdong Provincial People's Hospital, Guangdong Academy of Medical Sciences, Guangzhou, China; ^3^Department of Hematology, The Seventh Affiliated Hospital, Sun Yat-Sen University, Shenzhen, China; ^4^Medical Research Center, Guangdong Provincial People's Hospital, Guangdong Academy of Medical Sciences, Guangzhou, China

## Abstract

**Background:**

Pancreatic neuroendocrine neoplasm (pNEN), with the lowest 5-year survival rates in neuroendocrine tumors (NETs), exerts great threat to human health. Because large-scale population research aimed at pNEN is rare, we aimed to explore the tendencies and differences of changes in incidences and survival rates of pNEN in each decade from 1987 to 2016 and evaluate the impacts of age, sex, race, socioeconomic status (SES), and grade.

**Methods:**

Data on pNEN cases from 1987 to 2016 were extracted from the Surveillance, Epidemiology, and End Results Program (SEER) database. Kaplan–Meier, Cox proportional hazards regression analyses, and relative survival rates (RSRs) were used to identify risk factors for pNEN.

**Results:**

The incidence and survival duration of pNEN increase every decade due to medical developments. The disparities of long-term survival in different age, sex, and grade groups expanded over time while that in race and SES groups narrowed. Older age and higher grade are independent risk factors for poorer survival. Females have lower incidence and longer survival than males. Prognosis of Black patients and poor (medium and high poverty) patients improved.

**Conclusions:**

This study depicted changes in incidence and survival rates of pNEN over the past three decades and evaluated potential risk factors related to pNEN, benefiting future prediction of vulnerable and clinical options.

## 1. Introduction

Pancreatic neuroendocrine neoplasm (pNEN), previously termed “islet cell tumor,” is an infrequent and sporadic tumor, with an annual incidence of less than 1 case per 100,000 individuals. It currently accounts for 7% of all neuroendocrine tumors (NETs). Notably, in gastroenteropancreatic neuroendocrine tumors (GEP-NETs), pNEN has the lowest 5-year survival rates [1]. The World Health Organization 2017 classified pNEN into two main types, namely, well-differentiated neoplasms (defined as NETs) and poorly differentiated neuroendocrine carcinomas (defined as NECs). Clinically, pNENs can also be classified according to hormonal secretion into either functioning (F-pNENs) or nonfunctioning (NF-pNENs) depending on the presence of a syndrome related to atypical hormone secretion, such as hypoglycemia caused by abnormal insulin secretion [2].

The incidence of NETs has been on the rise in the USA over the past decades [3]. Recent studies indicated that the prognoses and overall survivals of NET patients are correlated to age and race [4–6]. However, only a few comprehensive analyses on pNEN in a large population are currently available, hence the need for more studies to benefit epidemiologic exploration and help find new therapeutic strategies. Pathological analysis of the grade of differentiation might contribute to prognostic assessment and therapy selection [7]. Socioeconomic status (SES), which is related to many malignancies, was reported to be associated with the financial ability of patients to shoulder the cost burden of effective specific diagnosis and timely medical inferences [8]. Therefore, to depict the overall incidence and survival trends and investigate pNEN-associated factors, we analyzed variables such as age, sex, race, SES, and pathological grade based on the SEER database.

## 2. Materials and Methods

### 2.1. Data Selection

The data on pNEN patients from 9 original SEER over 3 decades (1987–2016) were collected from the *SEER∗* Stat software program (version 8.3.5; National Cancer Institute, Bethesda, MD), which captures about 400,000 cancer cases annually and stores cancer data on approximately 30% of the U.S. population. The International Classification of Diseases for Oncology (3rd editions) (ICD-O-3) codes were used to identify primary locations of tumors of pancreas: C25.0-C25.9. The following ICD-O-3 codes of histology/behavior were defined as pNEN: pancreatic endocrine tumor, malignant (8150/3); insulinoma, malignant (8151/3); glucagonoma, malignant (8152/3); gastrinoma, malignant (8153/3); mixed pancreatic endocrine and exocrine tumor, malignant (8154/3); vipoma, malignant (8155/3); somatostatinoma, malignant (8156/3); carcinoid tumor (8240/2); enterochromaffin cell carcinoid (8241/3); enterochromaffin-like cell tumor, malignant (8242/3); goblet cell carcinoid (8243/3); mixed adenoneuroendocrine carcinoma (8244/3); adenocarcinoid tumor (8245/3); neuroendocrine carcinoma (8246); and atypical carcinoid tumor (8249/3). Patients diagnosed as pNEN from 1987 to 2016 with active follow-up were included and with active certificate or autopsy excluded.

### 2.2. Variable Definition

Sex, race, age, SES, and grade were the patient variables examined in this study. SES is divided into three levels: low poverty (0–9.9%), medium poverty (10–19.9%), and high poverty (≥20.0%). The medium- and high-poverty groups are integrated into one group in all analyses. SEER histologic grade information was used to classify cases: G1, well-differentiated; G2, moderately-differentiated; G3, poorly differentiated; G4, undifferentiated or anaplastic. G3 and G4 were combined into 1 category in this study.

### 2.3. Statistical Analysis

Data of incidences and relative survival rates (RSRs) on pNEN patients were divided into 3 decades of the study period and analyzed. The 12-month, 60-month, and 120-month RSRs were demonstrated by survival rate curves. The survival disparities in different patient stratifications were analyzed by Kaplan–Meier analysis with log-rank test. However, when analyzing race, only Whites and Blacks were included because of heterogeneity in racial composition. All statistical tests were two-sided, and the significance level was set at 0.05. Performed by Cox proportional hazards regression modeling, all five variables were included as covariates in multivariate analysis to evaluate contributions to pNEN, protective or risky. Hazard ratios and corresponding 95% confidence intervals (95%*CI*) were calculated for variables and adjusted for all listed covariates of interest. The Stata/SE software program (version 25.0; Stata Corp, College Station, TX) was used for data analysis.

## 3. Results

### 3.1. Incidence of pNEN Patients in 1987–2016

**Figure 1:**
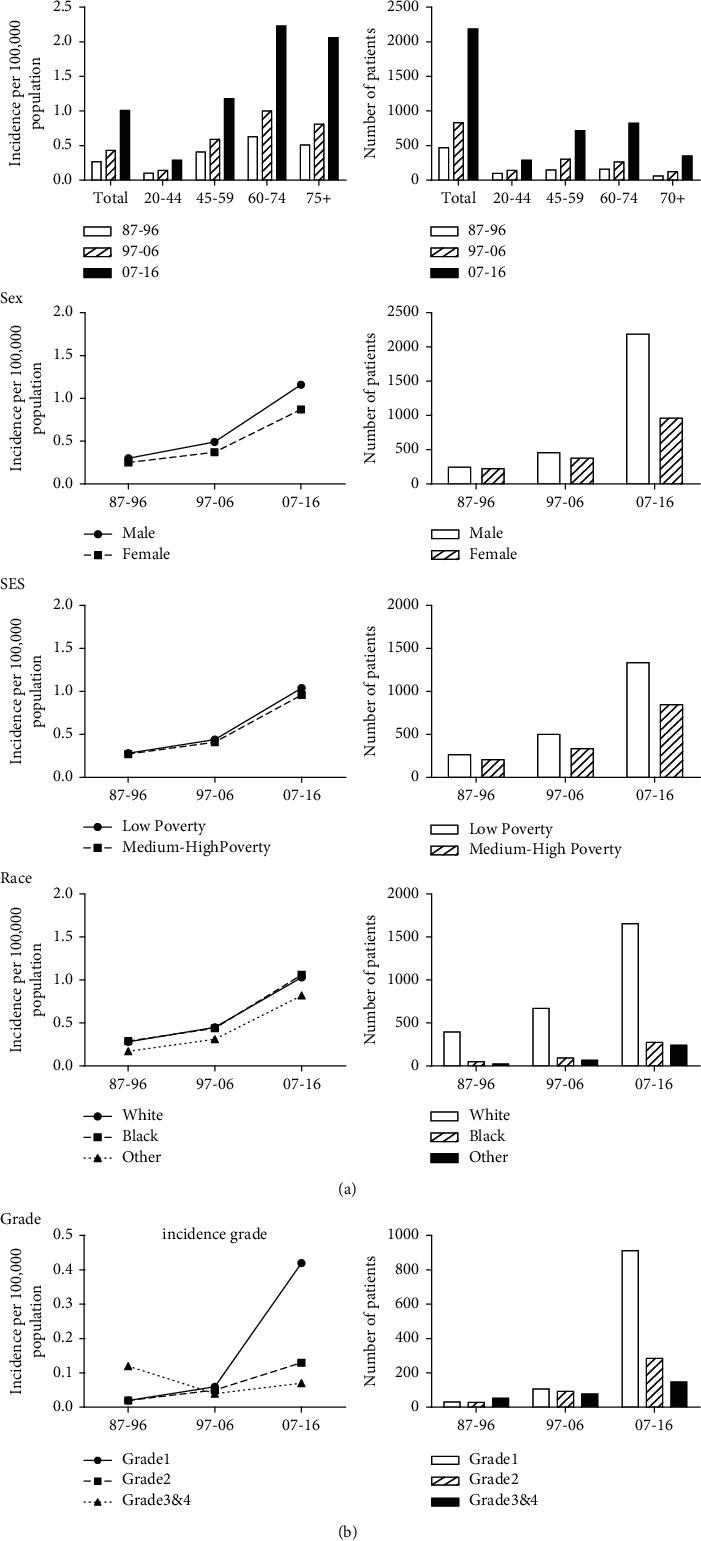
Analysis of incidence (per 100,000) and specific patient numbers of patients with pancreatic neuroendocrine neoplasm at 9 SEER sites according to age, sex, SES, race, and grade in 1987–2016.

The incidence data on pNEN over 3 decades of the study are extracted from the original nine registry sites in the SEER database maintained by the National Cancer Institute. In order to minimize the statistical influence of case number on incidences, the 0–20 age group was removed for small number (below 0.01 per 100,000), resulting in 3,491 cases for analysis. The total incidence of pNEN increased stably (from 0.27 to 0.43 and to 1.01). The similar trend can be found in all groups of variables, except G2 group (Figure 1 and Supplementary Table 1).

In age groups, the incidence rate and patient number of 60–74 age group were nearly the highest over the 3 decades (from 0.63 to 1.00 to 2.23). The incidence rate and patient number were higher in male patients, and in the third decade, the number of male patients was twice females. In race groups, the incidence of White patients and Black patients was close but the patient numbers of them were of great difference. The low poverty group showed a little higher incidence rate and case number than medium-high-poverty group. We in extra analyzed the race distribution by SES and the proportion of poor patients and rich patients in different races was nearly stable. The percentage of rich patients fluctuated between 60% and 65% over 30 years in Whites, which in Blacks was on the contrary. The incidence of G3&G4 group declined and then raised but the patient number stably increased over 3 decades.

The incidences of common tumors such as lung cancer and breast cancer remain stable, though diagnostic methods and individualized treatment options developed. However, that of pancreatic neuroendocrine tumor still maintains upward trend, suggesting that medical study should pay more attention on this low-incidence tumor to prevent epidemiological expand. As for linked risk factors in this study, females are more susceptible to environmental, hormonal, and genetic factors biologically, resulting in munity of carcinogen than males. Disparities of economic situation also exert influences on distinction of races. As there are developments and changes of consensus classification of pNEN, the proportion of G1 and G2 groups keeps sharply expanding since 1987.

### 3.2. Survival of pNEN Patients in 1987–2016

**Figure 2:**
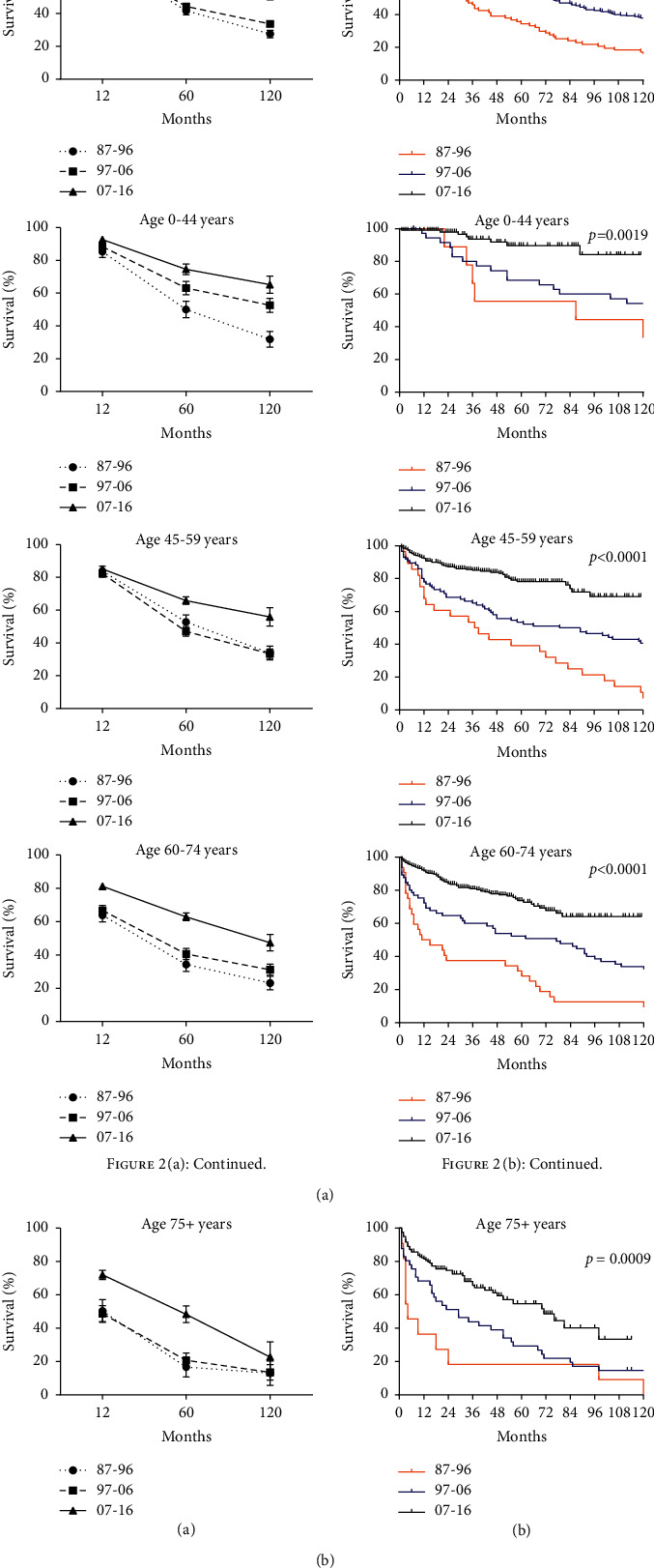
Trends in relative survival rate (a) and Kaplan–Meier survival curves (b) for patients with pNEN at 9 SEER sites according to age group (total and 0–44, 45–59, 60–74, and 75+ years) in 1987–1996, 1997–2006, and 2007–2016.

Data on 3466 pNEN cases were extracted and analyzed over the 3 decades from 9 SEER registry sites, respectively. The uptrend of RSRs could be found in all age groups, especially the overall 120-month RSR (from 27.5% to 33.6% to 51.1%) (Table 1). The gap between 60-month RSRs and 120-month RSRs expanded over time, except 75+ age group (Figure 2 and Supplementary Table 2).

**Figure 3:**
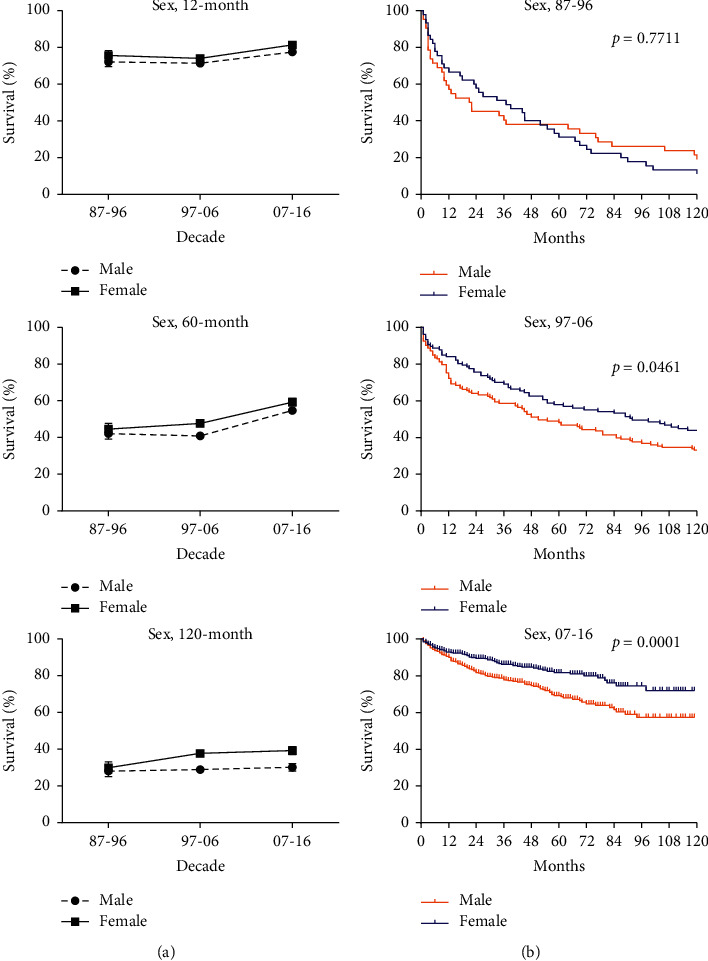
Trends in relative survival rate (a) and Kaplan–Meier survival curves (b) for patients with pNEN at 9 SEER sites according to sex group (male and female) in 1987–1996, 1997–2006, and 2007–2016.

RSRs in 12, 60, and 120 months were higher slightly in female patients. The sex disparity in the second and third decades was of significance in the study (*p* < 0.001 in 1997–2006 and *p*=0.0001 in 2007–2016). However, the 12-month RSRs of 60–74 age group according to sex in the second decade were significantly higher than that in the first decade. When studying 60- and 120-month RSRs, sex disparities in all age groups were found insignificant. In all, the significant rise of overall survival rates might benefit from group concern and medical improvement (Figure 3 and Supplementary Table 3).

### 3.3. Survival of pNEN in Different Race, SES, and Grade Groups

In race groups, RSRs in White patients were on a stable rise from 1987 to 2016, not always keeping above that in Black patients. RSRs of Blacks declined in the second decade and then increased. However, disparities of RSRs between two races were hardly found significant in the first decade, except for 45–59 age group (31.2% vs. 59.5%, *p* < 0.01). In the second decade, the 12-month RSR of 75+ age group in Whites was markedly higher than in Blacks (51.8% vs. 16.6%, *p* < 0.01). In the third decade, significant increase was suggested in 12-month relative survival rate in 60–74 year age group of White patients (82.0% vs. 74.4%, *p* < 0.01) (Supplementary Figure 1 and Supplementary Table 4).

In SES groups, low poverty group showed higher RSRs than medium-high-poverty group over the 3 decades, which was nearly demonstrated in all age groups according to SES. The similar situation was indicated in the second decade, except 0–44 year group. In the third decade, 60- and 120-month RSRs in low poverty group were higher than in medium-high-poverty group, respectively (65.6% vs. 59.8%, *p* < 0.00001, and 57.0% vs. 41.8%, *p* < 0.001) (Supplementary Figure 2 and Supplementary Table 5).

**Figure 4:**
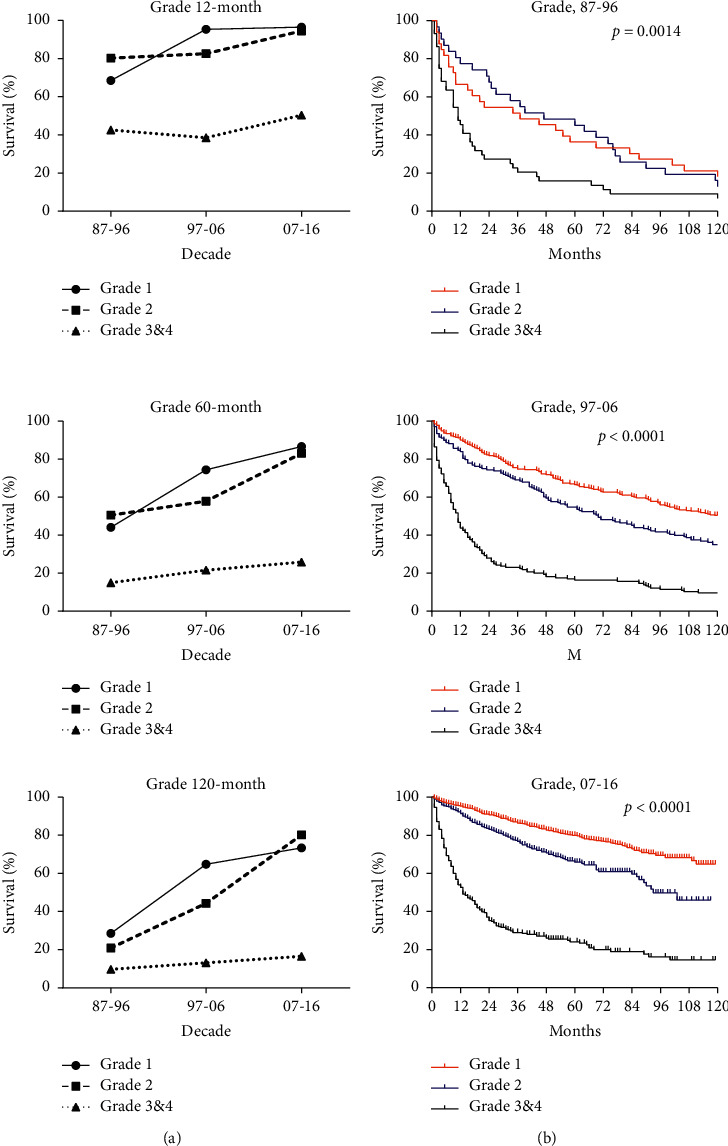
Trends in relative survival rate (a) and Kaplan–Meier survival curves (b) for patients with pNEN at 9 SEER sites according to grade group (grade 1, grade 2, and grade 3&4) in 1987–1996, 1997–2006, and 2007–2016.

The disparities of long-term survival rates among G1, G2, and G3&4 groups significantly enlarged over the study period (*p*=0.0014 in 1987–1996, *p* < 0.0001 in 1997–2006, and *p* < 0.0001 in 2007–2016) (Supplementary Table 6). Among grade groups, G3&4 group was always associated with the lowest RSRs. The gap between G1 group and G2 group widened in 1997–2006 and then narrowed in 2007–2016. In the first and the last decades, RSRs at any age of G1 and G2 groups were close while that of G3&4 group were significantly lower than the former two (Figure 4).

Hazard ratios for pNEN patients in Cox regression analysis according to age (*p*=0.007, *p* < 0.001, and *p* < 0.001 in 1987–2016) and grade (*p*=0.022, *p* < 0.001, and *p* < 0.001 in 1987–2016) were always greater than 1 over the 3 decades, which suggested that old age and high histological grade were related with short survival duration as independent predictors. Race was only significant statistically in the second decade (*p*=0.001, HR 0.024, 95% CI 1.067–2.499), so was SES in the third decade (*p*=0.023, HR 1.344, 95% CI 1.041–1.736) (Table 2). The risk of sex and SES appeared over time, especially in multivariate effects. Notably, G3&4 group (pNEC) with lower incidence demonstrated bare improvement of survival over 30 years while therapeutic effect of low-grade pNET was in satisfactory condition, suggesting necessity of clinical and medical scientists for further concern and advanced therapeutic technologies in this field.

## 4. Discussion

In this population-based study, both the incidence and RSRs (relative survival rates) of pNEN increased in each decade from 1987 to 2016, especially in the last decade. Although the survival disparities between different stratifications narrowed over time, the 10-year RSRs of pNEN patients with poor differentiation remained extraordinarily low, from 9.7% to 16.5% over 30 years.

We found that incidence of patients with pNEN demonstrated a sharp growth over the past 3 decades, especially in the last decade, from 0.27 to 1.01, a 3.7-fold increase. This might be related to the new classification set by the WHO for neuroendocrine cancer of the digestive system from 2000–2017. In recent years, the detection rate and diagnostic accuracy of pNEN improved as more imaging modalities emerged, such as PET with Ga-labelled somatostatin analogs [9] and endoscopic ultrasonography [10, 11]. The increased public health awareness also sensitized patients to the possible early signs of pNEN. Patients with functional pNEN were also more prone to seek medical treatments due to the presentation of symptoms caused by excessive hormone secretion. However, nonfunctional tumors accounting for over 70% (the proportion is still growing) of pNEN are often discovered at a later period presenting immunohistochemical positive for hormones due to the nonspecific symptoms linked to the tumor mass. [12–14].

The long-term survival rates showed a similar trend. Notably, the 120-month RSRs in 2007–2016 were almost double that of in 1987–1996 at different age stratifications. Similarly, for incidence, RSRs in the third decade were markedly elevated, indicating more effective therapies, more accurate histological assessment, better patient compliance, and the advent of new clinical biomarkers [15]. Furthermore, further studies involving genetic materials such as circulating tumor cells and microRNAs have broadened early diagnostic means and greatly improved targeted therapies. [15, 16]. Surgery is a curative treatment for primary pNEN, associated with longer survival, that can help alleviate symptoms caused by local mass and abnormal hormone secretion. [17–19] On the other hand, chemotherapy regimens such as somatostatin analogs are the first-line treatment for patients unable to undergo surgical resection. [7, 17]. However, both modalities do not rule out the possibility of disease recurrence.

In our study, multivariate Cox regression analysis showed that older age and higher grade were independent risk factors. Patients ≥60 years old had remarkably higher incidence and worse survival than other age groups, which might be due to the poorer average health and tolerance to medical interventions. Compared with young adults, older people are more prone to suffer from underlying diseases such as hypertension and diabetes mellitus. Moreover, they are more likely to develop tumors, suffer from progression, and even metastasis of tumors. A previous study suggested that timely primary site surgery (PSS) was an independent protective factor that benefited the overall survival of pNEN patients with liver metastases, the most common metastatic site, and cause of death in pNEN patients. However, other researchers revealed that elderly patients with poor differentiation are unsuitable for PSS. For those with low grade, age limitation remains. Therefore, whether elderly patients should accept PSS remains controversial [20–22]. We found no statistical significance in the improvement of 10-year RSR in pNEN patients aged ≥75 in 1987–2016, which is in line with another study outcome which showed that PSS or no PSS exerts scarcely influence on long-term survival [20]. Besides, another study found that the perioperative mortality of elderly patients with pNEN was higher [23].

We found that tumor grade is the most significant prognostic factor affecting survival and medical options. With case number increasing, the incidence of G1 pNEN increased most steeply (from 0.02 per 100,000 to 0.42 per 100,000 over time), possibly owing to further recognition and adoption of nomenclature, grading, and early diagnosis [12]. On top of that, current standardized pathology reports ensure a correct diagnosis and optimal treatment decision [24]. Some studies thought that CT and MRI had the potential of predicting the grade of pNEN according to tumor size, margin status, vascular invasion, tumor blood flow, and texture parameters [25–29]. Moreover, different grading systems may lead to inevitable nonconformity, puzzling clinical diagnosis, and therapeutic decisions for G1 and G2. Most pNENs are nonfunctional and prone to progression and metastasis if the diagnosis is delayed [12, 30]. In our study, the hazard ratios of grade were the highest among selected factors in Cox regression analysis. Well-differentiated G3 pNET and poorly differentiated pNEC were considered similar in terms of tumor grading. Therefore, the specific classification of G3 pNENs might greatly affect subsequent therapy or prognosis. However, we cannot exclude that the possibility of transformation from G3 pNET to pNEC is neglected.

Despite higher incidence in male patients versus female patients (1 : 0.75–0.95 in 1987–2016), the overall survival time in female patients was longer. Due to the development of abdominal imaging technologies, the incidental detection rate of nonfunctional pNEN, which is more common in females, has increased considerably, allowing for timely surgical treatment [31]. Due to the development of abdominal imaging technologies, the incidental detection rate of nonfunctional pNEN, which is more common in females, has increased considerably, allowing for timely surgical treatment.

White patients accounted for the vast majority of cases, whose incidences over the three decades were similar with Black patients. We further analyzed the race distribution in different SES that the average economic level of White patients was much higher than that of Black patients as expected. Marriage status was reported to be associated with cancer survival, mainly because of the linkage factors behind. Among races, the marriage rate of Blacks was the lowest. The number of unmarried Black patients is more than married ones. The latter have higher income levels, more health insurance investment, more surgical acceptance, lower mortality rate, and less distant metastases, depending on the financial situation [32, 33]. However, survival gaps between White and Black patients have narrowed over time, which may be attributed to the positive effects of an improving public health care. A higher rate of tumor metastasis in White patients might also be the origin of the offset in survival difference [6].

Nevertheless, there were some limitations in our study. Firstly, the sample size was relatively small compared due to the rarity of pNEN cases. Secondly, some prognostic factors reported potential association, such as primary site, stage, functional status, and tumor size, but were not involved in this analysis. Thirdly, clinical misclassification might mislead optimal therapies. Fourthly, not all histological types of pNEN were integrated into the SEER database, and some data of pNEN patients were not registered, which might result in a deviation between theoretical outcomes and actual situations.

## 5. Conclusion

Herein, we depicted the overall trend of pNEN in terms of prevalence, survival time, and related risk factors from 1987 to 2016 based on the SEER database. With the advent of various diagnostic means and treatment options in recent decades, incidences and survival rates have increased significantly. Age and tumor grade were found to be independent risk factors, with patients aged 60–74 years old and low-grade tumors having a greater incidence. Female patients had a lower incidence and longer survival compared to male patients. Race and SES were closely related, affecting early diagnosis and treatment decisions. Survival disparities between patient groups are shrinking, confirming the improvement of public health care in the U.S. This study may strengthen the knowledge of clinicians faced with patients of similar characteristics and might help predict the future trends of pNEN, as well as therapeutic schemes to balance survival disparities confirmed between age, sex, SES, race, and grade.

## Figures and Tables

**Table 1:** Relative survival rates of pancreatic neuroendocrine neoplasm patients during the periods of 1987–1996, 1997–2006, and 2007–2016 at nine SEER sites. Data are represented as mean ± standard error of the mean, with number of patients in parentheses.

Age group	Decade		
	1987–1996	1997–2006	2007–2016

12-Mo RS			
All	73.0 ± 2.1 (474)	73.7 ± 1.6 (833)	82.6 ± 0.9 (2159)^*∗∗∗∗*^
0–44	85.3 ± 3.5 (101)	88.4 ± 2.7 (146)	92.7 ± 1.6 (301)
45–59	83.8 ± 3.1 (149)	82.3 ± 2.2 (303)	85.1 ± 1.4 (706)
60–74	63.8 ± 3.9 (162)	66.7 ± 3.0 (264)	81.2 ± 1.5 (809)^*∗∗∗∗*^
75+	50.3 ± 6.8 (62)	48.7 ± 4.8 (120)	72.0 ± 2.8 (343)^*∗∗∗∗*^
60-Mo RS			
All	41.6 ± 2.4 (474)	44.3 ± 1.8 (833)	63.4 ± 1.5 (2159)^*∗∗∗∗*^
0–44	50.0 ± 5.0 (101)	63.1 ± 4.1 (146)^*∗*^	74.5 ± 3.3 (301)^*∗*^
45–59	52.8 ± 4.2 (149)	47.0 ± 3.0 (303)	65.8 ± 2.3 (706)^*∗∗∗∗*^
60–74	34.3 ± 4.1 (162)	40.6 ± 3.3 (264)	62.9 ± 2.4 (809)^*∗∗∗∗*^
75+	16.7 ± 6.0 (62)	20.7 ± 4.5 (120)	48.4 ± 5.0 (343)^*∗∗*^
120-Mo RS			
All	27.5 ± 2.3 (474)	33.6 ± 1.8 (833)^*∗*^	51.1 ± 2.8 (2159)^*∗∗∗*^
0–44	31.9 ± 4.8 (101)	52.6 ± 4.3 (146)^*∗∗*^	65.2 ± 5.2 (301)
45–59	33.8 ± 4.2 (149)	33.4 ± 2.9 (303)	55.9 ± 5.6 (706)^*∗*^
60–74	23.1 ± 4.0 (162)	31.1 ± 3.4 (264)	47.4 ± 4.9 (809)
75+	13.0 ± 7.3 (62)	13.5 ± 4.6 (120)	22.7 ± 9.0 (343)

Abbreviations: Mo, month; RS, relative survival; SEM, standard error of the mean. ^*∗*^*p* < 0.05, ^*∗∗*^*p* < 0.001, ^*∗∗∗*^*p* < 0.0001, ^*∗∗∗∗*^*p* < 0.00001 for comparisons with the preceding decade.

**Table 2:** Summary data for Cox regression analysis of survival in patients with pNEN from 1987 to 2016 at nine SEER sites.

Variable	Relative risk (95% CI)	*p* value

All 1987–1996		
Univariate		
Sex	1.035 (0.678–1.582)	0.873
Age	1.030 (1.013–1.047)	0.001
Race	1.144 (0.527–2.481)	0.734
SES	1.173 (0.749–1.836)	0.486
Grade	1.576 (1.194–2.080)	0.001
Multivariate		
Age	1.024 (1.006–1.041)	0.007
Grade	1.404 (1.051–1.877)	0.022
All 1997–2006		
Univariate		
Sex	0.714 (0.528–0.966)	0.029
Age	1.030 (1.019–1.042)	<0.001
Race	1.633 (1.067–2.499)	0.024
SES	1.112 (0.823–1.502)	0.490
Grade	1.904 (1.573–2.305)	<0.001
Multivariate		
Sex	0.856 (0.631–1.169)	0.333
Age	1.034 (1.023–1.046)	<0.001
Race	2.064 (1.331–3.198)	0.001
Grade	1.920 (1.582–2.330)	<0.001
All 2007–2016		
Univariate		
Sex	0.603 (0.462–0.786)	<0.001
Age	1.042 (1.032–1.053)	<0.001
Race	0.960 (0.658–1.400)	0.832
SES	1.344 (1.041–1.736)	0.023
Grade	2.584 (2.206–3.027)	<0.001
Multivariate		
Sex	0.578 (0.442–0.754)	<0.001
Age	1.036 (1.026–1.047)	<0.001
SES	1.440 (1.114–1.862)	0.005
Grade	2.400 (2.045–2.817)	<0.001

Abbreviations: 95% CI, 95% confidence interval; SES, socioeconomic status.

## Data Availability

The underlying data supporting this research could be found in *SEER∗* Stat software program.

## Supplementary Materials

Supplementary Materials The supplementary materials are divided into two parts: figures and tables. The supplementary figures show trends of incidence and survival curves of pNEN patients in race and SES groups (Supplementary Figures 1 and 2). The supplementary tables demonstrate all statistical data of incidence and RSRs according to studied variables (Supplementary Tables 1–6).
