# Supplementary material for: Trends in Incidence and Survival of Patients with Pancreatic Neuroendocrine Neoplasm, 1987–2016
Source: J Oncol. 2021 Dec 22;2021:4302675. doi: 10.1155/2021/4302675 (PMC8716229; doi:10.1155/2021/4302675)
Supplement: Supplementary Materials — The supplementary materials are divided into two parts: figures and tables. The supplementary figures show trends of incidence and survival curves of pNEN patients in race and SES groups (Supplementary Figures 1 and 2). The supplementary tables demonstrate all statistical data of incidence and RSRs according to studied variables (Supplementary Tables 1–6). [file 4302675.f1.zip › 4302675.f1/supple.figure.2.pdf]

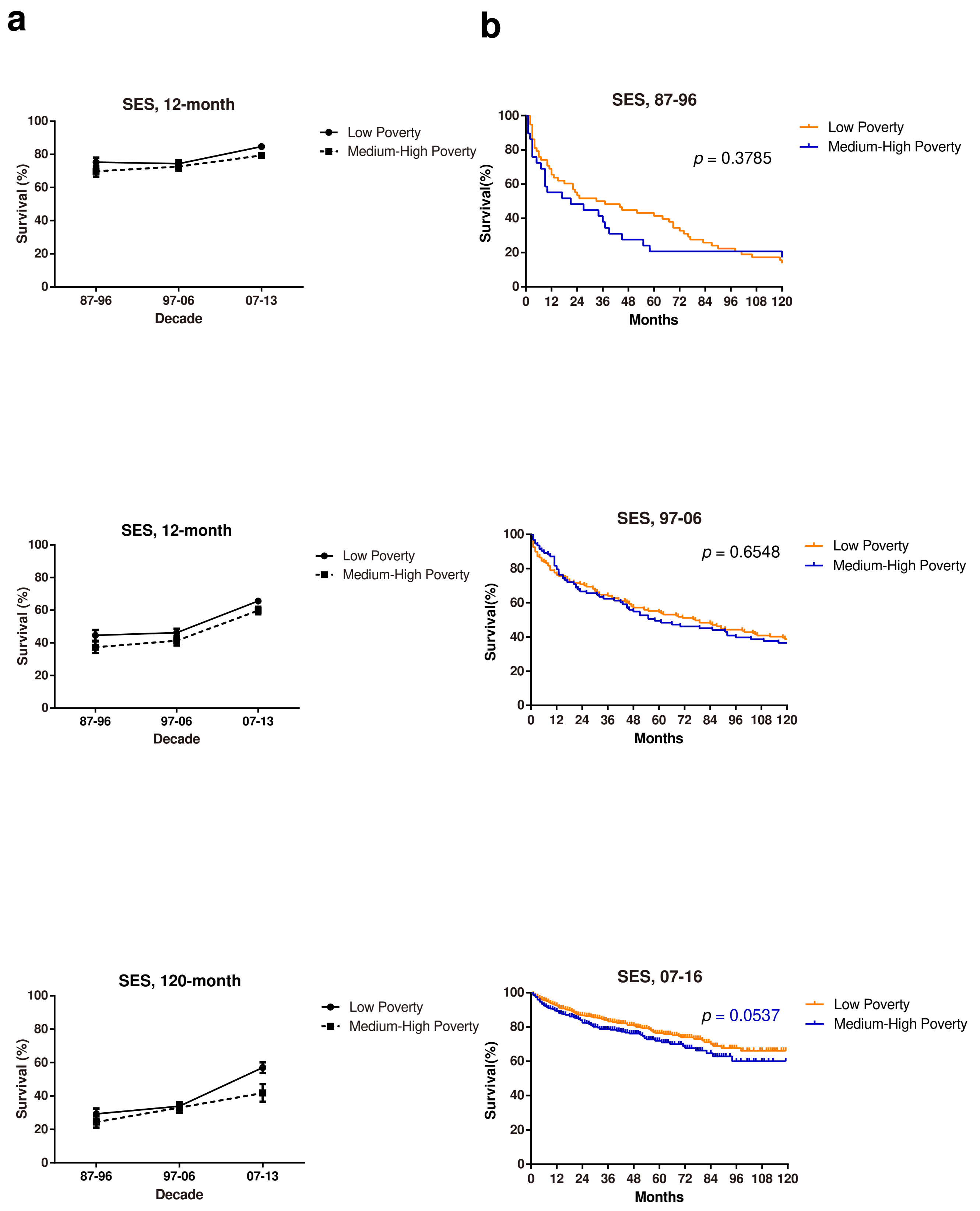

Supplementary Figure.2 Trends in relative survival rate **(a)** and Kaplan-Meier survival curves **(b)** for patients with pNEN at 9 SEER sites according to SES group (low poverty and medium-high poverty) in 1987-1996, 1997-2006, and 2007-2016.
