# Supplementary material for: Trends in Incidence and Survival of Patients with Pancreatic Neuroendocrine Neoplasm, 1987–2016
Source: J Oncol. 2021 Dec 22;2021:4302675. doi: 10.1155/2021/4302675 (PMC8716229; doi:10.1155/2021/4302675)
Supplement: Supplementary Materials — The supplementary materials are divided into two parts: figures and tables. The supplementary figures show trends of incidence and survival curves of pNEN patients in race and SES groups (Supplementary Figures 1 and 2). The supplementary tables demonstrate all statistical data of incidence and RSRs according to studied variables (Supplementary Tables 1–6). [file 4302675.f1.zip › 4302675.f1/Supplementary Table 1.docx]

**Supplementary Table S1**. The incidence of pNEN according to age group and decade within sex, SES, grade and race groups from 1987 to 2016 at the nine original SEER sites. Data are incidence per 100,000 people by year of diagnosis, with the number of patients in parentheses.

|  | | Age  Groups | Decade | | |
| --- | --- | --- | --- | --- | --- |
| Variable | |  | 1987-1996 | 1997-2006 | 2007-2016 |
| Total |  |  |  |  |  |
|  |  | 20-75+ | 0.27（472） | 0.43（833） | 1.01（2186） |
|  |  | 20-44 | 0.10（98） | 0.14（140） | 0.29（291） |
|  |  | 45-59 | 0.41（150） | 0.59（305） | 1.18（715） |
|  |  | 60-74 | 0.63（162） | 1.00（264） | 2.23（827） |
|  |  | 75+ | 0.51（62） | 0.81（124） | 2.06（353） |
| Sex | Male |  |  |  |  |
|  |  | 20-75+ | 0.30（246） | 0.49（458） | 1.16（1222） |
|  |  | 20-44 | 0.08（38） | 0.13（65） | 0.27（138） |
|  |  | 45-59 | 0.47（83） | 0.69（175） | 1.41（420） |
|  |  | 60-74 | 0.82（95） | 1.28（157） | 2.69（472） |
|  |  | 75+ | 0.78（66） | 1.06（61） | 2.83（192） |
|  | Female |  |  |  |  |
|  |  | 20-75+ | 0.25（226） | 0.37（375） | 0.87（964） |
|  |  | 20-44 | 0.12（38） | 0.15（75） | 0.30（153） |
|  |  | 45-59 | 0.36（67） | 0.49（130） | 0.95（295） |
|  |  | 60-74 | 0.48（67） | 0.76（157） | 1.81（355） |
|  |  | 75+ | 0.41（32） | 0.66（63） | 1.55（161） |
| Race | white |  |  |  |  |
|  |  | 20-75+ | 0.28（396） | 0.45（671） | 1.03（1656） |
|  |  | 20-44 | 0.10（75） | 0.15（114） | 0.27（196） |
|  |  | 45-59 | 0.42（125） | 0.60（244） | 1.17（537） |
|  |  | 60-74 | 0.66（142） | 0.98（210） | 2.18（638） |
|  |  | 75+ | 0.51（54） | 0.79（103） | 2.04（285） |
|  | Black |  |  |  |  |
|  |  | 20-75+ | 0.29（50） | 0.44（94） | 1.06（274） |
|  |  | 20-44 | 0.15（17） | 0.12（14） | 0.34（48） |
|  |  | 45-59 | 0.48（16） | 0.71（38） | 1.41（101） |
|  |  | 60-74 | 0.65（13） | 1.25（28） | 2.74（96） |
|  |  | 75+ | 0.52（4） | 1.41（14） | 2.36（29） |
|  | Other |  |  |  |  |
|  |  | 20-75+ | 0.17（26） | 0.31（67） | 0.82（243） |
|  |  | 20-44 | 0.06（6） | 0.09（11） | 0.28（44） |
|  |  | 45-59 | 0.29（9） | 0.42（23） | 0.94（72） |
|  |  | 60-74 | 0.34（7） | 0.94（26） | 2.08（90） |
|  |  | 75+ | 0.55（4） | 0.54（7） | 1.91（37） |
| SES | Low Poverty |  |  |  |  |
|  |  | 20-75+ | 0.28（264） | 0.44（499） | 1.04（1336） |
|  |  | 20-44 | 0.09（48） | 0.16（90） | 0.29（169） |
|  |  | 45-59 | 0.46（95） | 0.56（173） | 1.19（440） |
|  |  | 60-74 | 0.61（86） | 1.10（168） | 2.32（513） |
|  |  | 75+ | 0.51（35） | 0.74（68） | 2.1806（214） |
|  | Medium/High Poverty |  |  |  |  |
|  |  | 20-75+ | 0.27（207） | 0.41（334） | 0.96（848） |
|  |  | 20-44 | 0.11（49） | 0.16（50） | 0.28（121） |
|  |  | 45-59 | 0.35（55） | 0.63（132） | 1.15（275） |
|  |  | 60-74 | 0.66（76） | 0.87（96） | 2.08（313） |
|  |  | 75+ | 0.50（27） | 0.90（56） | 2.06（139） |
| Grade | G1 |  |  |  |  |
|  |  | 20-75+ | 0.02（31） | 0.06（107） | 0.42（912） |
|  |  | 20-44 | 0.01（9） | 0.02（24） | 0.15（156） |
|  |  | 45-59 | 0.03（10） | 0.08（42） | 0.49（296） |
|  |  | 60-74 | 0.04（10） | 0.10（27） | 0.93（344） |
|  |  | 75+ | 0.02（2） | 0.09（14） | 0.68（116） |
|  | G2 | 20-75+ | 0.02（29） | 0.05（93） | 0.13（285） |
|  |  | 20-44 | 0.01（5） | 0.03（27） | 0.04（42） |
|  |  | 45-59 | 0.03（11） | 0.06（32） | 0.16（95） |
|  |  | 60-74 | 0.04（10） | 0.08（21） | 0.28（1 per05） |
|  |  | 75+ | 0.02（3） | 0.08（13） | 0.25（43） |
|  | G3&G4 | 20-75+ | 0.12（54） | 0.04（78） | 0.07（149） |
|  |  | 20-44 | 0.002（2） | 0.01（11） | 0.01（11） |
|  |  | 45-59 | 0.03（10） | 0.05（26） | 0.07（44） |
|  |  | 60-74 | 0.05（14） | 0.11（29） | 0.17（62） |
|  |  | 75+ | 0.08（10） | 0.08（12） | 0.19（32） |

Abbreviation: SES, socioeconomic status.
