# Supplementary material for: Trends in Incidence and Survival of Patients with Pancreatic Neuroendocrine Neoplasm, 1987–2016
Source: J Oncol. 2021 Dec 22;2021:4302675. doi: 10.1155/2021/4302675 (PMC8716229; doi:10.1155/2021/4302675)
Supplement: Supplementary Materials — The supplementary materials are divided into two parts: figures and tables. The supplementary figures show trends of incidence and survival curves of pNEN patients in race and SES groups (Supplementary Figures 1 and 2). The supplementary tables demonstrate all statistical data of incidence and RSRs according to studied variables (Supplementary Tables 1–6). [file 4302675.f1.zip › 4302675.f1/Supplementary Table 2.docx]

**Supplementary Table 2**. Relative survival rates of patients with pNEN during the periods of 1987-1996, 1997-2006, and 2007-2016 at nine SEER sites. Data are mean ± standard error of the mean, with number of patients in parentheses.

|  |  | **Decade** |  |
| --- | --- | --- | --- |
| **Age Group** | **1987-1996** | **1997-2006** | **2007-2016** |
| 12-Mo RS |  |  |  |
| All | 73.0 ± 2.1 (474) | 73.7 ± 1.6 (833) | 82.6 ± 0.9 (2159)**** |
| 0-44 | 85.3 ± 3.5 (101) | 88.4 ± 2.7 (146) | 92.7 ± 1.6 (301) |
| 45-59 | 83.8 ± 3.1 (149) | 82.3 ± 2.2 (303) | 85.1 ± 1.4 (706) |
| 60-74 | 63.8 ± 3.9 (162) | 66.7 ± 3.0 (264) | 81.2 ± 1.5 (809)**** |
| 75+ | 50.3 ± 6.8 (62) | 48.7 ± 4.8 (120) | 72.0 ± 2.8 (343)**** |
| 60-Mo RS |  |  |  |
| All | 41.6 ± 2.4 (474) | 44.3 ± 1.8 (833) | 63.4 ± 1.5 (2159)**** |
| 0-44 | 50.0 ± 5.0 (101) | 63.1 ± 4.1 (146)* | 74.5 ± 3.3 (301)* |
| 45-59 | 52.8 ± 4.2 (149) | 47.0 ± 3.0 (303) | 65.8 ± 2.3 (706)**** |
| 60-74 | 34.3 ± 4.1 (162) | 40.6 ± 3.3 (264) | 62.9 ± 2.4 (809)**** |
| 75+ | 16.7 ± 6.0 (62) | 20.7 ± 4.5 (120) | 48.4 ± 5.0 (343)** |
| 120-Mo RS |  |  |  |
| All | 27.5 ± 2.3 (474) | 33.6 ± 1.8 (833)* | 51.1 ± 2.8 (2159)*** |
| 0-44 | 31.9 ± 4.8 (101) | 52.6 ± 4.3 (146)** | 65.2 ± 5.2 (301) |
| 45-59 | 33.8 ± 4.2 (149) | 33.4 ± 2.9 (303) | 55.9 ± 5.6 (706)* |
| 60-74 | 23.1 ± 4.0 (162) | 31.1 ± 3.4 (264) | 47.4 ± 4.9 (809) |
| 75+ | 13.0 ± 7.3 (62) | 13.5 ± 4.6 (120) | 22.7 ± 9.0 (343) |

Abbreviations: Mo, month; RS, relative survival; SEM, standard error of the mean.

**p* < 0.05, ***p* < 0.001, and ****p* < 0.0001 for comparisons with the preceding decade.
