# Supplementary material for: Trends in Incidence and Survival of Patients with Pancreatic Neuroendocrine Neoplasm, 1987–2016
Source: J Oncol. 2021 Dec 22;2021:4302675. doi: 10.1155/2021/4302675 (PMC8716229; doi:10.1155/2021/4302675)
Supplement: Supplementary Materials — The supplementary materials are divided into two parts: figures and tables. The supplementary figures show trends of incidence and survival curves of pNEN patients in race and SES groups (Supplementary Figures 1 and 2). The supplementary tables demonstrate all statistical data of incidence and RSRs according to studied variables (Supplementary Tables 1–6). [file 4302675.f1.zip › 4302675.f1/Supplementary Table 3.docx]

**Supplementary Table 3**. 12-month, 60-month and 120-month relative survival rates of pNEN patients according to sex, age group, and calendar period from 1987 to 2016 at nine SEER sites. Data are means ± standard error of the mean, with number of patients in parentheses.

|  |  | **Sex** | | | |
| --- | --- | --- | --- | --- | --- |
| **Decade** | **Age Group** | **Male** | **Female** | | |
| 87-96 | 12-Mo RS |  | |  |  |
|  | All | 71.1± 3.0 (247) | | 75.0 ± 3.0 (227) |  |
|  | 0-44 | 87.4 ± 5.4 (39) | | 84.0 ± 4.7 (62) |  |
|  | 45-59 | 88.6 ±3.6 (83) | | 77.6 ± 5.2 (66) |  |
|  | 60-74 | 55.2 ±5.3 (95) | | 75.9 ± 5.4 (67)** |  |
|  | 75+ | 49.8 ± 9.7 (30) | | 50.1 ± 9.4 (32) |  |
|  | 60-Mo RS |  | |  |  |
|  | All | 40.5 ± 3.4 (247) | | 42.6 ± 3.5 (227) |  |
|  | 0-44 | 41.7 ± 8.0 (39) | | 55.2 ± 6.4 (62) |  |
|  | 45-59 | 58.8± 5.6 (83) | | 45.1 ± 6.3 (66) |  |
|  | 60-74 | 29.1 ± 5.1 (95) | | 41.1 ± 6.5 (67) |  |
|  | 75+ | 20.3 ± 9.5 (30) | | 12.5 ± 6.9 (32) |  |
|  | 120-Mo RS |  | |  |  |
|  | All | 27.7 ± 3.3 (247) | | 27.2 ± 3.2 (227) |  |
|  | 0-44 | 31.3 ± 7.7(39) | | 32.4 ± 6.1 (62) |  |
|  | 45-59 | 33.8 ± 4.2 (83) | | 28.9 ± 5.8 (66) |  |
|  | 60-74 | 18.1 ± 4.9 (95) | | 29.9 ± 6.5 (67) |  |
|  | 75+ | 20.3 ± 9.5 (30) | | 0.0 ± 0.0 (32)* |  |
| 97-06 | 12-Mo RS |  | |  |  |
|  | All | 71.0 ± 2.2 (461) | | 76.9± 2.2 (372) |  |
|  | 0-44 | 81.5 ± 4.7 (70) | | 94.8 ± 2.6 (76)* |  |
|  | 45-59 | 80.5 ± 3.0 (175) | | 84.7 ± 3.2 (128) |  |
|  | 60-74 | 61.6 ± 4.0 (157) | | 73.8 ± 4.4 (107)* |  |
|  | 75+ | 54.2 ± 6.9 (59) | | 43.2 ± 6.6 (61) |  |
|  | 60-Mo RS |  | |  |  |
|  | All | 41.8 ± 2.5 (461) | | 47.3 ± 2.7 (372) |  |
|  | 0-44 | 58.8 ± 6.0 (70) | | 67.1 ± 5.5 (76) |  |
|  | 45-59 | 44.4 ± 3.9 (175) | | 50.4 ± 4.5 (128) |  |
|  | 60-74 | 37.5 ± 4.3 (157) | | 45.0 ± 5.2 (107) |  |
|  | 75+ | 22.1 ± 6.8 (59) | | 17.8 ± 5.5 (61) |  |
|  | 120 Mo RS |  | |  |  |
|  | All | 30.6 ± 2.5 (461) | | 37.2 ± 2.8 (372) |  |
|  | 0-44 | 46.5 ± 6.2 (70) | | 58.2 ± 5.8 (76) |  |
|  | 45-59 | 30.0 ± 3.7 (175) | | 37.8 ± 4.5 (128) |  |
|  | 60-74 | 29.6 ± 4.5 (157) | | 32.7 ± 5.3 (107) |  |
|  | 75+ | 11.7 ± 6.6 (59) | | 13.7 ± 5.9 (61) |  |
|  |  |  | |  |  |
| 07-16 | 12-Mo RS |  | |  |  |
|  | All | 81.4 ± 1.2 (1212) | | 84.2 ± 1.3 (947) |  |
|  | 0-44 | 89.8 ± 2.6 (144) | | 95.6 ± 1.8 (157) |  |
|  | 45-59 | 84.5 ± 1.9 (416) | | 85.8 ± 2.1 (290) |  |
|  | 60-74 | 79.2 ± 2.0 (463) | | 83.9 ± 2.1 (346) |  |
|  | 75+ | 73.0 ± 3.8 (189) | | 70.7 ± 4.1 (154)* |  |
|  | 60-Mo RS |  | |  |  |
|  | All | 60.8 ± 2.0 (1212) | | 66.9 ± 2.1 (947) |  |
|  | 0-44 | 70.5 ± 4.8 (144) | | 78.8 ± 4.3 (157) |  |
|  | 45-59 | 62.4 ± 3.1 (416) | | 70.9 ± 3.4 (290) |  |
|  | 60-74 | 60.3 ± 3.3 (463) | | 66.4 ± 3.6 (346) |  |
|  | 75+ | 48.0 ± 7.1 (189) | | 47.0 ± 6.9 (154) |  |
|  | 120 Mo RS |  | |  |  |
|  | All | 51.1 ± 2.8 (1212) | | 54.5 ± 4.8 (947) |  |
|  | 0-44 | 53.3 ± 7.1 (144) | | 83.3 ± 3.6 (157) |  |
|  | 45-59 | 58.4 ± 4.5 (416) | | 55.8 ± 9.3 (290) |  |
|  | 60-74 | 42.6 ± 6.6 (463) | | 51.8 ± 6.7 (346) |  |
|  | 75+ | 21.5 ± 13.9 (189) | | 18.8 ± 12.0 (154) |  |

Abbreviations: Mo, month; RS, relative survival; SEM, standard error of the mean.

**p* < 0.01, ***p* < 0.001, and ****p* < 0.0001
