# Supplementary material for: Trends in Incidence and Survival of Patients with Pancreatic Neuroendocrine Neoplasm, 1987–2016
Source: J Oncol. 2021 Dec 22;2021:4302675. doi: 10.1155/2021/4302675 (PMC8716229; doi:10.1155/2021/4302675)
Supplement: Supplementary Materials — The supplementary materials are divided into two parts: figures and tables. The supplementary figures show trends of incidence and survival curves of pNEN patients in race and SES groups (Supplementary Figures 1 and 2). The supplementary tables demonstrate all statistical data of incidence and RSRs according to studied variables (Supplementary Tables 1–6). [file 4302675.f1.zip › 4302675.f1/Supplementary Table 4 (1).docx]

**Supplementary Table 4**. 12-month, 60-month and 120-month relative survival rates of pNEN patients according to race, age group, and calendar period from 1987 to 2016 at nine SEER sites. Data are means ± standard error of the mean, with number of patients in parentheses.

|  |  | **Race** | | |
| --- | --- | --- | --- | --- |
| **Decade** | **Age Group** | **White** | **Black** | **Other** |
| 87-96 | 12-Mo RS |  |  |  |
|  | All | 73.7 ± 2.3 (399) | 72.5 ± 6.6 (49) | 62.4 ± 9.7 (26) |
|  | 0-44 | 88.6 ± 3.6 (78) | 82.5 ± 9.3 (17) | 50.0 ± 20.4 (6) |
|  | 45-59 | 85.3 ± 3.2 (125) | 80.3 ± 10.4 (15) | 66.9 ± 15.8 (9) |
|  | 60-74 | 62.6 ± 4.2 (142) | 63.4 ± 13.9 (13) | 86.4 ± 13.3 (7) |
|  | 75+ | 53.7 ± 7.3 (54) | 25.4 ± 22.0 (4) | 26.3 ± 22.8 (4) |
|  | 60-Mo RS |  |  |  |
|  | All | 41.1 ± 2.6 (399) | 48.2 ± 7.6 (49) | 35.7 ± 9.6 (26) |
|  | 0-44 | 51.7 ± 5.7 (78) | 41.7 ± 12.1 (17) | 50.0 ± 20.4 (6) |
|  | 45-59 | 52.1 ± 4.6 (125) | 62.3 ± 13.1 (15) | 34.1 ± 16.1 (9) |
|  | 60-74 | 33.4 ± 4.3 (142) | 44.5 ± 15.6 (13) | 30.1 ± 18.0 (7) |
|  | 75+ | 16.4 ± 6.3 (54) | 25.4 ± 22.0 (4) | 0.0 ± 0.0 (4) |
|  | 120 Mo RS |  |  |  |
|  | All | 25.9 ± 2.5 (399) | 37.4 ± 8.1 (49) | 28.3 ± 9.1 (26) |
|  | 0-44 | 32.0 ± 5.4 (78) | 24.3 ± 10.6 (17) | 50.0 ± 20.4 (6) |
|  | 45-59 | 31.2 ± 4.4 (125) | 59.5 ± 14.5 (15)* | 22.7 ± 14.2 (9) |
|  | 60-74 | 22.5 ± 4.2 (142) | 22.4 ± 14.5 (13) | 30.1 ± 18.0 (7) |
|  | 75+ | 9.8 ± 6.8 (54) | 25.4 ± 22.0 (4) | 0.0 ± 0.0 (4) |
| 97-06 | 12-Mo RS |  |  |  |
|  | All | 75.6 ± 1.7 (670) | 64.2 ± 5.0 (95)* | 67.5 ± 5.8 (67) |
|  | 0-44 | 89.8 ± 2.8 (118) | 87.7 ± 8.3 (16) | 72.8 ± 13.4 (11) |
|  | 45-59 | 83.5 ± 2.4 (242) | 77.0 ± 7.0 (38) | 78.5 ± 8.6 (23) |
|  | 60-74 | 69.3 ± 3.3 (210) | 54.4 ± 9.6 (28) | 57.5 ± 9.9 (26) |
|  | 75+ | 51.8 ± 5.3 (100) | 16.6 ± 10.8 (13)* | 57.9 ± 18.9 (7) |
|  | 60-Mo RS |  |  |  |
|  | All | 45.8 ± 2.1 (670) | 34.9 ± 5.2 (95) | 40.8 ± 6.2 (67) |
|  | 0-44 | 62.7 ± 4.5 (118) | 63.3 ± 12.2 (16) | 63.7 ± 14.5 (11) |
|  | 45-59 | 50.7 ± 3.3 (242) | 27.5 ± 7.5 (38)** | 39.6 ± 10.3 (23) |
|  | 60-74 | 41.4 ± 3.7 (210) | 38.4 ± 9.7 (28) | 33.5 ± 9.7 (26) |
|  | 75+ | 20.9 ± 5.0 (100) | 8.8 ± 8.4 (13) | 31.7 ± 18.9 (7) |
|  | 120 Mo RS |  |  |  |
|  | All | 33.6 ± 2.1(670) | 31.0 ± 5.2 (95) | 34.0 ± 6.1 (67) |
|  | 0-44 | 52.1 ± 4.8 (118) | 50.9 ± 12.7 (16) | 55.0± 15.1 (11) |
|  | 45-59 | 34.6 ± 3.2 (242) | 24.8 ± 7.2 (38) | 31.3 ± 9.8 (23) |
|  | 60-74 | 29.6 ± 3.8 (210) | 38.4 ± 9.7 (28) | 30.2 ± 9.6 (26) |
|  | 75+ | 14.3 ± 4.8 (100) | 0.0 ± 0.0 (13) | 17.8 ± 16.4 (7) |
| 07-16 | 12-Mo RS |  |  |  |
|  | All | 82.6 ± 0.9 (2159) | 80.3 ± 2.6 (273) | 81.6 ± 2.7 (243) |
|  | 0-44 | 93.4 ± 1.9 (199) | 97.9 ± 2.1 (50) | 84.0 ± 5.6 (49)* |
|  | 45-59 | 85.6± 1.6 (529) | 81.0 ± 4.1 (101) | 85.8 ± 4.5 (71) |
|  | 60-74 | 82.0 ± 1.7 (625) | 74.4 ± 4.9 (94)* | 82.0 ± 4.4 (87) |
|  | 75+ | 72.5 ± 3.1 (277) | 66.3 ± 9.4 (28) | 68.2 ± 8.2 (36) |
|  | 60-Mo RS |  |  |  |
|  | All | 63.4 ± 1.5 (2159) | 61.2 ± 4.2 (273) | 66.1 ± 4.3 (243) |
|  | 0-44 | 75.1 ± 3.9 (199) | 71.4 ± 8.2 (50) | 74.1 ± 8.8 (49) |
|  | 45-59 | 66.2 ± 2.7 (529) | 57.2 ± 7.0 (101) | 71.4 ± 6.5 (71) |
|  | 60-74 | 62.4 ± 2.7 (625) | 65.6 ± 7.4 (94) | 62.5 ± 8.6 (87) |
|  | 75+ | 47.8 ± 5.6 (277) | 41.4 ± 13.2 (28) | 50.4 ± 10.5 (36) |
|  | 120-Mo RS |  |  |  |
|  | All | 51.1 ± 2.8 (2159) | 54.9 ± 6.9 (273) | 59.1 ± 11.0 (243) |
|  | 0-44 | 63.5 ± 6.6 (2159) | 65.1 ± 10.7 (50) | 74.0 ± 8.8 (49) |
|  | 45-59 | 60.9 ± 5.5 (529) | 50.0 ± 11.5 (101) | 29.1 ± 23.2 (71) |
|  | 60-74 | 44.3 ± 5.2 (625) | 51.3 ± 13.8 (94) | 63.8 ± 8.7 (87) |
|  | 75+ | 16.4 ± 9.3 (277) | 37.2 ± 14.5 (28) | 0.0 ± 0.0 (36)** |

Abbreviations: Mo, month; RS, relative survival; SEM, standard error of the mean.

**p* < 0.01, ***p* < 0.001, and ****p* < 0.0001 for comparisons with the preceding group.
