# Supplementary material for: Trends in Incidence and Survival of Patients with Pancreatic Neuroendocrine Neoplasm, 1987–2016
Source: J Oncol. 2021 Dec 22;2021:4302675. doi: 10.1155/2021/4302675 (PMC8716229; doi:10.1155/2021/4302675)
Supplement: Supplementary Materials — The supplementary materials are divided into two parts: figures and tables. The supplementary figures show trends of incidence and survival curves of pNEN patients in race and SES groups (Supplementary Figures 1 and 2). The supplementary tables demonstrate all statistical data of incidence and RSRs according to studied variables (Supplementary Tables 1–6). [file 4302675.f1.zip › 4302675.f1/Supplementary Table 5.docx]

**Supplementary Table 5**. 12-month, 60-month and 120-month relative survival rates of pNEN patients according to SES, age group, and calendar period from 1987 to 2016 at nine SEER sites. Data are means ± standard error of the mean, with number of patients in parentheses.

|  |  | **SES** | | | |
| --- | --- | --- | --- | --- | --- |
| **Decade** | **Age Group** | **Low Poverty** | **Medium-High Poverty** | | |
| 87-96 | 12-Mo RS |  | |  |  |
|  | All | 75.3 ± 2.7 (266) | | 69.8 ± 3.3 (207) |  |
|  | 0-44 | 84.1 ± 5.2 (50) | | 86.1 ± 4.9 (50) |  |
|  | 45-59 | 83.6 ± 3.9 (95) | | 83.9 ± 5.1 (54) |  |
|  | 60-74 | 67.8 ± 5.2 (86) | | 59.3 ± 5.8 (76) |  |
|  | 75+ | 58.5 ± 9.1 (35) | | 39.4 ± 9.9 (27) |  |
|  | 60-Mo RS |  | |  |  |
|  | All | 44.6 ± 3.3 (266) | | 37.3 ± 3.6 (207) |  |
|  | 0-44 | 54.6 ± 7.1 (50) | | 44.4 ± 7.1 (50) |  |
|  | 45-59 | 56.5 ± 7.3 (95) | | 46.3 ± 7.0 (54) |  |
|  | 60-74 | 35.0 ± 5.6 (86) | | 33.1 ± 5.9 (76) |  |
|  | 75+ | 16.1 ± 7.6 (35) | | 16.9 ± 9.2 (27) |  |
|  | 120-Mo RS |  | |  |  |
|  | All | 29.3 ± 3.2 (266) | | 24.4 ± 3.4 (207) |  |
|  | 0-44 | 40.3 ± 7.2 (50) | | 22.5 ± 6.0 (50) |  |
|  | 45-59 | 34.5 ± 5.2 (95) | | 31.7 ± 6.8 (54) |  |
|  | 60-74 | 22.8 ± 5.3 (86) | | 23.0 ± 5.8 (76) |  |
|  | 75+ | 7.4 ± 7.3 (35) | | 16.9 ± 9.2 (27) |  |
| 97-06 | 12-Mo RS |  | |  |  |
|  | All | 74.4 ± 2.0 (497) | | 72.6 ± 2.5 (336) |  |
|  | 0-44 | 92.4 ± 2.8 (92) | | 81.6 ± 5.3 (54)* |  |
|  | 45-59 | 82.4 ± 2.9 (172) | | 82.2 ± 3.4 (131) |  |
|  | 60-74 | 65.8 ± 3.8 (168) | | 67.9 ± 4.9 (96) |  |
|  | 75+ | 48.6 ± 6.5 (65) | | 48.5 ± 7.2 (55) |  |
|  | 60-Mo RS |  | |  |  |
|  | All | 46.2 ± 2.4 (497) | | 41.3 ± 2.9 (336) |  |
|  | 0-44 | 66.2 ± 5.1 (92) | | 57.9 ± 6.8 (54) |  |
|  | 45-59 | 51.5 ± 3.9 (172) | | 41.0 ± 4.4 (131) |  |
|  | 60-74 | 39.8 ± 4.1 (168) | | 41.8 ± 5.5 (96) |  |
|  | 75+ | 18.2 ± 5.6 (65) | | 23.1 ± 7.0 (55) |  |
|  | 120 Mo RS |  | |  |  |
|  | All | 33.9 ± 2.4 (497) | | 33.0 ± 2.9 (336) |  |
|  | 0-44 | 53.6 ± 5.4 (92) | | 51.0 ± 6.9 (54) |  |
|  | 45-59 | 36.9 ± 3.9 (172) | | 28.4 ± 4.1 (131) |  |
|  | 60-74 | 27.6 ± 4.1 (168) | | 36.3 ± 6.0 (96) |  |
|  | 75+ | 11.0 ± 5.4 (65) | | 15.3 ± 7.4 (55) |  |
|  |  |  | |  |  |
| 07-16 | 12-Mo RS |  | |  |  |
|  | All | 84.7 ± 1.1 (1315) | | 79.4 ± 1.5 (842) |  |
|  | 0-44 | 91.4 ± 2.2 (175) | | 94.5 ± 2.2 (125) |  |
|  | 45-59 | 88.4 ± 1.6 (432) | | 79.7 ± 2.5 (274) |  |
|  | 60-74 | 82.8 ± 1.8 (501) | | 78.7 ± 2.5 (307) |  |
|  | 75+ | 75.5 ± 3.4 (207) | | 66.4 ± 4.6 (136) |  |
|  | 60-Mo RS |  | |  |  |
|  | All | 65.6 ± 1.9 (1315) | | 59.8 ± 2.3 (842)**** |  |
|  | 0-44 | 75.5 ± 4.1 (175) | | 72.8 ± 5.3 (125) |  |
|  | 45-59 | 67.7 ± 3.0 (432) | | 62.8 ± 3.7 (274) |  |
|  | 60-74 | 64.2 ± 3.1 (501) | | 60.5 ± 3.9 (307) |  |
|  | 75+ | 54.3 ± 6.8 (207) | | 36.9 ± 6.5 (136) |  |
|  | 120 Mo RS |  | |  |  |
|  | All | 57.0 ± 3.2 (1315) | | 41.8 ± 5.3 (842)** |  |
|  | 0-44 | 71.8 ± 5.9 (175) | | 51.6 ± 9.1 (125) |  |
|  | 45-59 | 63.2 ± 3.7 (432) | | 40.5 ± 9.4 (274)* |  |
|  | 60-74 | 46.9 ± 6.2 (501) | | 47.0 ± 5.8 (307) |  |
|  | 75+ | 22.1 ± 11.4 (207) | | 26.1 ± 14.7 (136) |  |

Abbreviations: Mo, month; RS, relative survival; SEM, standard error of the mean.

**p* < 0.05, ***p* < 0.001, and ****p* < 0.0001
