# Supplementary material for: Trends in Incidence and Survival of Patients with Pancreatic Neuroendocrine Neoplasm, 1987–2016
Source: J Oncol. 2021 Dec 22;2021:4302675. doi: 10.1155/2021/4302675 (PMC8716229; doi:10.1155/2021/4302675)
Supplement: Supplementary Materials — The supplementary materials are divided into two parts: figures and tables. The supplementary figures show trends of incidence and survival curves of pNEN patients in race and SES groups (Supplementary Figures 1 and 2). The supplementary tables demonstrate all statistical data of incidence and RSRs according to studied variables (Supplementary Tables 1–6). [file 4302675.f1.zip › 4302675.f1/Supplementary Table 6.docx]

**Supplementary Table 6**. 12-month, 60-month and 120-month relative survival rates of pNEN patients according to grade, age group, and calendar period from 1987 to 2016 at nine SEER sites. Data are means ± standard error of the mean, with number of patients in parentheses.

|  |  | **Grade** | | |
| --- | --- | --- | --- | --- |
| **Decade** | **Age Group** | **Grade 1** | **Grade 2** | **Grade3&4** |
| 87-96 | 12-Mo RS |  |  |  |
|  | All | 68.5 ± 8.5 (31) | 80.3 ± 7.6 (29) | 36.9 ± 8.2 (36)*** |
|  | 0-44 | 88.9 ± 10.5 (9) | 100.0 ± 0.0 (5) | 100.0 ± 0.0 (2) |
|  | 45-59 | 80.5 ± 12.7 (10) | 73.2 ± 13.5 (11) | 50.2 ± 15.9 (10) |
|  | 60-74 | 51.1 ± 16.2 (10) | 80.8 ± 12.8 (10) | 29.1 ± 12.3 (14)** |
|  | 75+ | 0.0 ± 0.0 (2) | 67.0 ± 27.3 (3) | 21.0 ± 13.3 (10) |
|  | 60-Mo RS |  |  |  |
|  | All | 44.1 ± 9.3 (31) | 50.5 ± 9.7 (29) | 15.0 ± 6.2 (36)** |
|  | 0-44 | 56.0 ± 14.7 (9) | 60.2 ± 22.0 (5) | 0.0 ± 0.0 (2) |
|  | 45-59 | 62.4 ± 16.1 (10) | 28.0 ± 13.8 (11) | 20.5 ± 13.0 (10) |
|  | 60-74 | 22.4 ± 14.2 (10) | 72.6 ± 15.0 (10)* | 14.9 ± 9.7 (14)** |
|  | 75+ | 0.0 ±0.0 (2) | 36.8 ± 30.1 (3) | 11.0 ± 10.5 (10) |
|  | 120 Mo RS |  |  |  |
|  | All | 28.5 ± 8.7 (31) | 20.9 ± 8.5 (34) | 9.7 ± 5.4 (36) |
|  | 0-44 | 45.4 ± 16.9 (9) | 60.2 ± 22.0 (5) | 0.0 ± 0.0 (2) |
|  | 45-59 | 22.1 ± 14.0 (10) | 0.0 ± 0.0 (11) | 10.4 ± 9.9 (10) |
|  | 60-74 | 22.4 ± 14.2 (10) | 23.1 ± 14.6 (10) | 7.9 ± 7.6 (14) |
|  | 75+ | 0.0 ± 0.0 (2) | 0.0 ± 0.0 (3) | 11.0 ± 10.5(10) |
| 97-06 | 12-Mo RS |  |  |  |
|  | All | 95.4 ± 2.2 (108) | 82.6 ± 4.1 (92)** | 43.3 ± 5.7 (79)**** |
|  | 0-44 | 96.0 ± 3.9 (25) | 85.3 ± 6.8 (27) | 82.7 ±11.3 (12) |
|  | 45-59 | 97.8 ± 2.4 (42) | 84.3 ± 6.6 (31)* | 42.6 ± 9.8 (26)*** |
|  | 60-74 | 96.6 ± 3.6 (27) | 87.0 ± 7.8 (21) | 38.3 ± 9.1 (29)*** |
|  | 75+ | 82.0 ± 11.4 (14) | 63.8 ± 14.0 (13) | 17.4 ± 11.2 (12)* |
|  | 60-Mo RS |  |  |  |
|  | All | 74.4 ± 4.7 (108) | 57.9 ± 5.5 (92)* | 21.6 ± 4.8 (79)**** |
|  | 0-44 | 76.6 ± 8.6 (25) | 67.2 ± 9.1 (27) | 55.4 ± 15.1 (12) |
|  | 45-59 | 81.1 ± 6.5 (42) | 49.5 ± 9.2 (31)** | 11.9 ± 6.5 (26)** |
|  | 60-74 | 77.2 ± 9.6 (27) | 66.9 ± 11.4 (21) | 25.7 ± 8.5 (29)** |
|  | 75+ | 38.2 ± 16.2 (14) | 40.6 ± 16.9 (13) | 0.0 ± 0.0 (12)* |
|  | 120 Mo RS |  |  |  |
|  | All | 64.8 ± 5.4 (108) | 44.3 ± 5.9 (92)* | 13.1 ± 4.1 (79)**** |
|  | 0-44 | 65.1 ± 9.8 (25) | 52.9 ± 9.8 (27) | 37.8 ± 15.0 (12) |
|  | 45-59 | 74.6 ± 7.7 (42) | 34.6 ± 9.0 (31)** | 20.7 ± 7.0 (35) |
|  | 60-74 | 63.0 ± 11.7 (27) | 53.1 ± 13.4 (21) | 16.4 ± 7.6 (29)* |
|  | 75+ | 21.7 ± 14.2 (14) | 28.3 ± 18.4 (13) | 0.0 ± 0.0 (12) |
| 07-16 | 12-Mo RS |  |  |  |
|  | All | 96.5 ± 0.7 (906) | 94.5 ± 1.6 (277) | 50.4 ± 4.5 (143)**** |
|  | 0-44 | 97.4 ± 1.4 (164) | 97.5 ± 2.6 (43) | 87.6 ± 11.7 (10) |
|  | 45-59 | 98.1 ± 0.9 (292) | 94.8 ± 2.9 (93) | 38.5 ± 8.0 (42)**** |
|  | 60-74 | 96.2 ± 1.3 (337) | 99.1 ± 1.5 (100) | 55.4 ± 6.9 (59)**** |
|  | 75+ | 90.3 ± 3.2 (113) | 77.3 ± 7.9 (41) | 46.1 ± 9.2 (32)* |
|  | 60-Mo RS |  |  |  |
|  | All | 86.6 ± 2.0 (906) | 83.0 ± 4.1 (277) | 25.9 ± 4.8 (143)**** |
|  | 0-44 | 89.6 ± 3.2 (164) | 94.6 ± 3.9 (43) | 23.5 ± 20.2 (10)**** |
|  | 45-59 | 87.2 ± 3.2 (292) | 84.2 ± 5.5 (93) | 21.1± 7.5 (42)**** |
|  | 60-74 | 86.2 ± 3.7 (337) | 83.0 ± 7.0 (100) | 30.4 ± 6.9 (59)**** |
|  | 75+ | 76.6 ± 7.7 (113) | 67.6 ± 15.9 (41) | 21.4 ± 11.6 (32)* |
|  | 120-Mo RS |  |  |  |
|  | All | 73.4 ± 4.8 (906) | 80.2 ± 4.9 (277) | 16.5 ± 7.1 (143)**** |
|  | 0-44 | 83.1 ± 5.6 (164) | 93.7 ± 4.4 (43) | 0.0 ± 0.0 (10)**** |
|  | 45-59 | 77.7 ± 8.1 (292) | 85.3 ± 5.5 (93) | 16.8 ± 7.3 (10)*** |
|  | 60-74 | 75.5 ± 7.4 (337) | 80.1 ± 8.7 (100) | 21.5 ± 7.3 (59)**** |
|  | 75+ | 51.3 ± 29.8 (113) | 52.2 ± 22.2 (41) | 16.4 ± 11.9 (32) |

Abbreviations: Mo, month; RS, relative survival; SEM, standard error of the mean.

**p* < 0.05, ***p* < 0.001, and ****p* < 0.0001 for comparisons with the former grade group.
